# α6 Integrin (α6^high^)/Transferrin Receptor (CD71)^low^ Keratinocyte Stem Cells Are More Potent for Generating Reconstructed Skin Epidermis Than Rapid Adherent Cells

**DOI:** 10.3390/ijms18020282

**Published:** 2017-01-27

**Authors:** Elodie Metral, Nicolas Bechetoille, Frédéric Demarne, Walid Rachidi, Odile Damour

**Affiliations:** 1 Gattefossé, 36 chemin de Genas, F-69800 Saint-Priest, France; metral.elodie@gmail.com (E.M.); nbechetoille@gattefosse.com (N.B.); fdemarne@gattefosse.com (F.D.); 2 Commissariat à l’énergie atomique et aux énergies alternatives (CEA)/Institut Nanosciences et cryogénie (INAC)/SYstèmes Moléculaires et nanoMatériaux pour l’Energie et la Santé (SyMMES)/Lésions des acides nucléiques (LAN), 17 avenue des martyrs, F-38054 Grenoble CEDEX, France; 3 Hospices Civils de LYON (HCL)/Banque de Tissus et Cellules/Laboratoire des Substituts Cutanés, 5 place d’Arsonval, F-69000 Lyon, France; odile.damour@chu-lyon.fr; 4 Department of Biological Sciences, University Grenoble Alpes, F-38000 Grenoble, France

**Keywords:** skin, epidermis, basal keratinocytes, keratinocyte stem cells, adhesion, α6 integrin, CD71

## Abstract

The epidermis basal layer is composed of two keratinocyte populations: Keratinocyte Stem cells (KSC) and Transitory Amplifying (TA) cells that arise from KSC division. Unfortunately, no specific marker exists to differ between KSC and TA cells. Here, we aimed at comparing two different methods that pretended to isolate these two populations: (i) the rapid adhesion method on coated substrate and (ii) the flow cytometry method, which is based on the difference in cell surface expressions of the α6 integrin and transferrin receptor (CD71). Then, we compared different parameters that are known to discriminate KSC and TA populations. Interestingly, we showed that both methods allow enrichment in stem cells. However, cell sorting by flow cytometry (α6^high^/CD71^low^) phenotype leads to a better enrichment of KSC since the colony forming efficiency is five times increased versus total cell suspension, whereas it is only 1.4 times for the adhesion method. Moreover, α6^high^/CD71^low^ cells give rise to a thicker pluristratified epithelium with lower seeding density and display a low Ki67 positive cells number, showing that they have reached the balance between proliferation and differentiation. We clearly demonstrated that cells isolated by a rapid adherent method are not the same population as KSC isolated by flow cytometry following α6^high^/CD71^low^ phenotype.

## 1. Introduction

Keratinocytes, the main cellular type of the epidermis, display different differentiation levels. Within the epidermal basal layer, keratinocyte stem cells (KSC) and their direct progeny called transitory amplifying (TA) cells are tightly anchored to the basement membrane and share similar morphological and biochemical aspects. Although both cell types exhibit distinct proliferative capacities, they ensure skin homeostasis and keratinocyte renewal throughout the lifetime of the organism. In suprabasal layers of epidermis, differentiated cells (DC) lose their proliferative capacity and migrate toward the stratum corneum to assure the barrier function of skin.

These different cellular populations (KSC, TA and DC) have their own properties. TA cells maintain the short-term renewal of the epidermis and KSC assure the renewal over the lifetime as shown by their persistence in the skin of aged people [1]. Indeed, KSC have an important division potential (>130 divisions) compared to TA cells, which have a limited number of divisions (<50 divisions) [2,3]. Activation of KSC proliferation, which is normally quiescent or slow-cycling cells, occurs to repair epidermis when it is exposed to extreme tissue damage induced by external physical or chemical factors such as UV or ionizing radiations, or, in the case of wounding (burning or cutting). In the stem cell niche, KSC, are quiescent in vivo [4,5,6] and are protected from apoptosis and differentiation stimuli, which could result in a premature depletion of the stem cell compartment [7]. The niche can be defined as a microenvironment in which stromal cell-derived factors modulate adhesive interactions, cell cycle regulation and intercellular signaling. At this level, integrins and transmembrane receptors play a key role in cell–cell and cell–extracellular matrix communication and are strongly implicated in regulation, maintenance and even identification of KSC [8,9,10,11,12,13,14,15,16]. Integrin–ligands such as fibronectin, collagens and laminins are the major constituents of the KSC niche [17].

As KSC are protected in their niche, they are more resistant to Ultraviolet B radiation (UVB) [18] and ionizing radiation [19,20] than TA being in proliferation state. Thus, since mutations can lead to their deregulation and skin cancer, it is important to be able to isolate this cell population from others (TA and DC) in order to perform biochemical, pharmaceutical and toxicological studies.

Since no unique specific markers for KSC have been identified so far, it is still hard to isolate them. However, techniques using one or several basal cell markers have been proposed to enrich keratinocyte population in basal cells (KSC and TA). For example, integrin α6-antibody coupled to fluorochrome or to magnetic beads allows the isolation of positive cells by flow cytometry [21] or under a magnetic field, respectively. Combination of β1-integrin and Rhodamine 123A [22], as well as β1-integrin and Desmoglein 3 [10], or β1-integrin and K1/K10 [23], would allow isolation of epidermal stem cells candidate. A cell population α6^high^/CD71^low^ sorted by flow cytometry [8] was also proposed to isolate KSC. Indeed, this population displays a quiescent state once extracted, a high clonogenic and proliferative potential once cultured [8] and a great ability to reconstruct an epidermis in vivo with a very low cellular seeding density [24]. In comparison, the α6^high^/CD71^high^ cells have characteristics close to those shared by TA cells. The importance of these adhesion molecules at the surface of basal keratinocytes would make them able to adhere quicker than the others, a characteristic which has been used as another approach to isolate them [14]. Thus, from a freshly extracted keratinocyte population, the adhesion time would be shorter for KSC than for other cells [15]. However, even if several authors have used this technique for studying KSC [14,25,26,27,28,29], the stem cell characteristics of these rapid adherent (RA) cells is still not proved. Currently, it has only been shown that RA cells are similar to α6+ [21] and β1+ [14] cells.

The aim of this paper is to elucidate whether RA cells isolated by the rapid adhesion method are similar to α6^high^/CD71^low^ cells sorted by flow cytometry. Therefore, after optimization of the rapid adhesion method, the RA cells were compared to α6^high^/CD71^low^ cells sorted by the flow cytometry method [8] using a Colony Forming Efficiency test (CFE%) as the first criterion [2], and their ability to reconstruct a human pluristratified epidermis (RHE) as the second criterion.

## 2. Results

### 2.1. Optimization of the Rapid Adhesion Method

**Figure 1:**
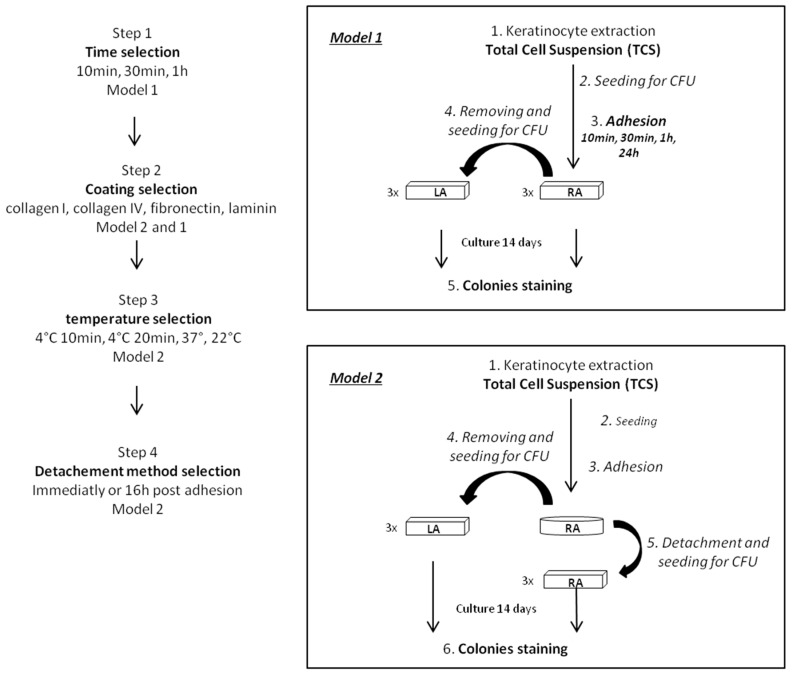
Schematic representation of experimental protocol of rapid adhesion method optimization using model 1 or model 2 according to the step. Arrows correspond to chronological steps.

By using two different models evaluating enrichment in KSC (Figure 1), one displaying the proportion of holoclones and meroclones directly in isolated cells (model 1) and the other defining the clonogenic potential of isolated cells colony forming efficiency (CFE) obtained with the same cellular density) (model 2), different parameters were compared in order to define the best conditions for obtaining the best KSC enrichment.

#### 2.1.1. The KSC Enrichment is Inversely Proportional to the Adhesion Time

**Figure 2:**
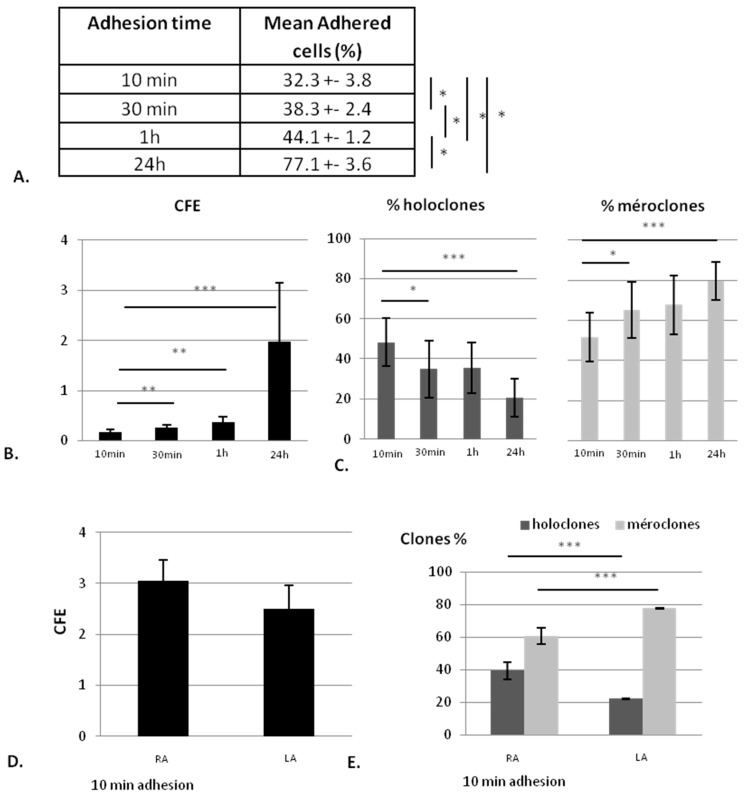
Influence of adhesion time on clonogenic potential of the different isolated cells (Rapid adherent (RA) and Low adherent (LA)). (**A**) percentage of adhered cells for each adhesion time; (**B**) colony-forming efficacy (CFE) obtained with different adhesion times (10 min, 30 min, 1 h and 24 h); (**C**) colony repartition (percentage of holoclones and meroclones composing colonies) from RA cells having adhered within different times; (**D**) CFE of RA cells (cells having adhered within 10 min) and LA cells; and (**E**) proportion of holoclones and meroclones containing in RA and LA cells, mean of three donors. *n* = 3 * data significantly different (*p* < 0.05), ** *p* < 0.005, *** *p* < 0.0005.

Model 1 shows that extracted keratinocytes adhere to a feeder layer composed of irradiated human fibroblasts according to the adhesion time. Indeed, the percentage of adhered cell number significantly increases with the adhesion time (Figure 2A) from 32% ± 3.8% for 10 min to 38% ± 2.4% for 30 min, 44% ± 1.2% for 1 h and 77% ± 3.6% for 24 h.

According to the percentage of adhered cells, the CFE increases with adhesion time (Figure 2B), but the proportion of holoclones (KSC) statistically decreases (*p* = 0.04 between 10 min and 30 min and 0.0005 between 10 min and 24 h), whereas the percentage of meroclones, derived from TA, increases (*p* = 0.04 between 10 min and 30 min and 0.0005 between 10 min and 24 h). However, the highest proportion of regular contour-holoclones, a signature of KSC, is found after 10 min of adhesion time even if CFE is the lowest (Figure 2C). Together, these results show that a short adhesion time allows for obtaining a cellular suspension richer in KSC (holoclones), whereas a longer adhesion time leads to more TA cells (meroclones).

Following these results, adhesion time of 10 min was chosen and two cell populations were defined: Rapid Adherent cells (RA) for cells that have adhered within 10 min and Low Adherent cells (LA) for the rest of the cells. After 10 min of adhesion, RA and LA display the same CFE (Figure 2D,E) in which the percentage of holoclones is higher in RA than in LA, demonstrating that RA population is significantly richer in KSC than LA, which is enriched in TA (*p* < 0.0001).

#### 2.1.2. Collagen Type I Leads to Maintenance of Clonogenic Capacity of Isolated Cells

**Figure A1:**
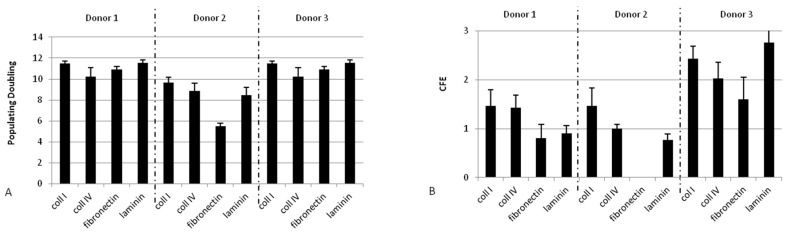
Influence of adhesion support on clonogenic and proliferative potential of obtained cellular suspension (RA and LA) obtained from a model 2 assay (**A**). Population doubling obtained after detachment of cells having adhered for 10 min to different supports; (**B**) CFE obtained after detachment of cells having adhered for 10 min to different supports. Collagen I presents the most positive and homogenous difference between RA and LA cells for the number of total colonies as well as for holoclone colonies and population doubling for the three donors studied. Results for three donors are separated by the dotted line.

Different coatings (collagen I, collagen IV, fibronectin and laminin) were compared using model 2 for CFE and populating doubling (PD) of each isolated RA from three donors (Figure A1). However, even if there is no significant difference between coatings, collagen I, which leads to both CFE and PD among the highest compared to those obtained with other coatings for the three donors tested, is then selected.

**Figure 3:**
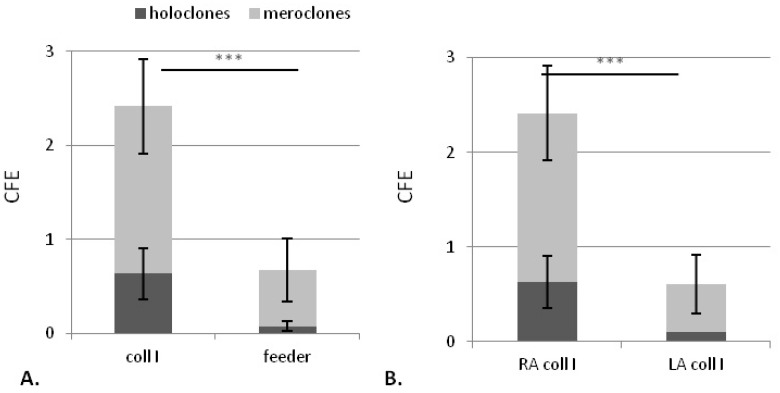
Influence of adhesion support on clonogenic potential of obtained cellular suspension (RA and LA) obtained from model 1 assay. (**A**) CFE obtained for RA after adhesion for 10 min on collagen I or on feeder layers; (**B**) CFE obtained for RA and LA cells after adhesion for 10 min on collagen I. Mean of three donors. *n* = 3. *** data significantly different *p* < 0.0005.

Figure 3 shows the comparison of RA on collagen I versus the human feeder layer using model 1. Both CFE and holoclone number are significantly higher for RA having adhered to collagen I compared to those adhered on feeder layer (*p* < 0.0001 for both parameters) (Figure 3A). Moreover, these two criteria are also significantly higher for RA on collagen I than for LA (*p* < 0.0001 for both parameters), confirming the improvement of adhesion step with collagen I compared to the feeder layer, a condition leading to similar RA and LA CFE (Figure 3B).

#### 2.1.3. Adhesion at 37 °C Leads to a Higher Clonogenic Potential of Isolated Cells

**Figure 4:**
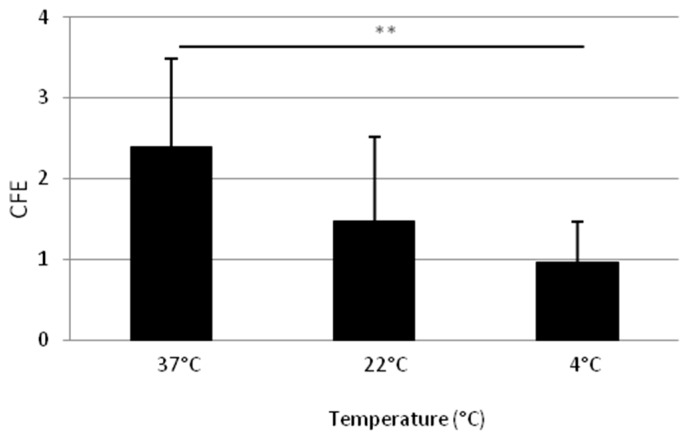
Influence of adhesion temperature on the clonogenic potential of isolated cellular suspension (RA and LA). CFE obtained for RA cells after detachment of cells having adhered for 10 min on collagen I at different temperature. Mean of three donors. *n* = 3. ** data significantly different *p* < 0.005.

Figure 4 shows the influence of temperature on KSC enrichment (model 2). Adhesion for 10 min at 37 °C allows a higher CFE than for 10 min at 4 °C (*p* = 0.004 and 0.0012, respectively) or at 22 °C (not statistically different but reproducible on three donors). Furthermore, 37 °C is then selected for the following steps.

#### 2.1.4. Isolated Cells Detached One Day after Adhesion with Accutase Display a Higher Clonogenic Potential

The detachment step was investigated by two parameters: the time after adhesion before detachment (immediately or 24 h post adhesion) and the dissociation reagent, trypsin or accutase, which is currently used for detaching embryonal stem cells (supplier claim).

**Figure 5:**
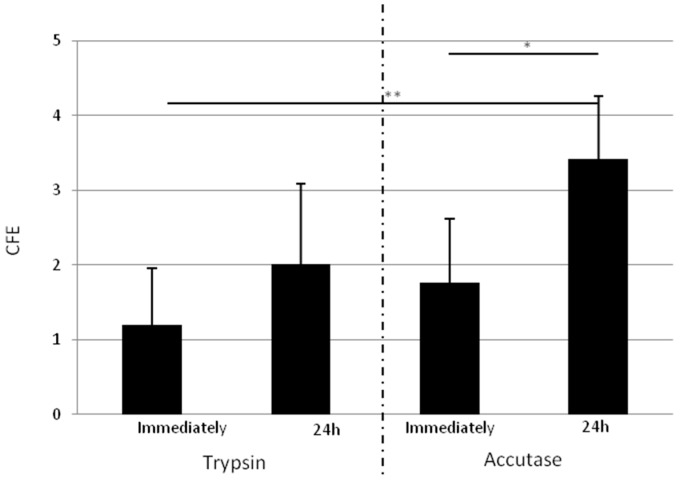
Influence of detachment method on clonogenic potential of RA cells. CFE obtained for RA cells immediately detached or 24 h after adhesion, with trypsin (TE) or accutase (Ac). Results obtained by the two dissociation reagents are separated by the dotted line. Detaching cells 24 h after adhesion with accutase allows for obtaining a better clone number. Mean of three donors. *n* = 3. * data significantly different (*p* < 0.05), ** *p* < 0.005.

Figure 5 compares the CFE obtained with RA immediately detached after the 10 min adhesion step or 24 h post adhesion, with trypsin or accutase. Regardless to the digestion reagent, the CFE appears more important for RA detached 24 h post adhesion for the three donors tested. Moreover, 24 h after adhesion, accutase allows for obtaining a significantly more important increase of CFE (*p* = 0.0018) than trypsin. Thus, the detachment method is optimized by the selection of accutase 24 h post adhesion.

### 2.2. Comparison of KSC Sorted by Flow Cytometry Following the α6^high^/CD71^low^ Phenotype to KSC Isolated by Rapid Adhesion Method (RA Cells)

**Figure 6:**
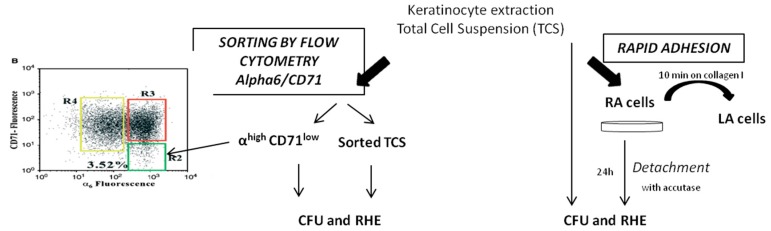
Experimental protocol of the comparison of the functional characterization of RA and the α^high^ CD71^low^ population. Arrows correspond to following steps.

From the same donor, cells were sorted by cytometry following the α6^high^/CD71^low^ phenotype and isolated by optimized rapid adhesion method and then qualified using CFE and capacity to generate reconstructed human epidermis (RHE) (Figure 6).

#### 2.2.1. KSC Sorted by Flow Cytometry (α6^high^/CD71^low^) Display a Better CFE Enrichment

**Figure 7:**
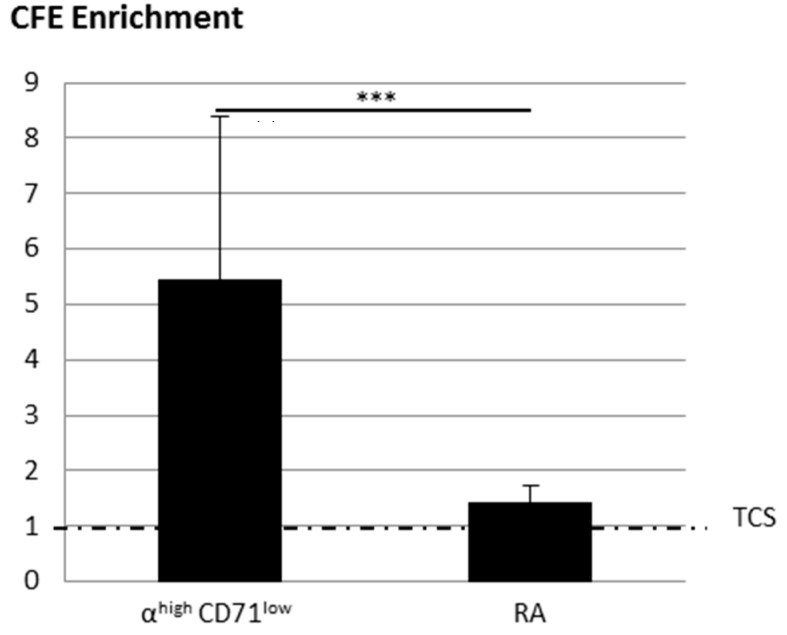
Influence of enrichment method (flow cytometry compared to optimized rapid adhesion method) of CFE enrichment. CFE enrichment obtained with α6^high^/CD71^low^ or RA cells compared to total cell suspension (TCS) (equal to 1). The dotted line corresponds to TCS (equal to 1). Mean of three samples of three donors ± SD. *** data significantly different *p* < 0.0005.

Figure 7 shows the improvement of the CFE of RA obtained by the optimized rapid adhesion method and KSC obtained with the cell sorting by flow cytometry (CSFC) method following the α6^high^/CD71^low^ phenotype compared to the associated total cell suspension (equal to 1). The CFE of α6^high^/CD71^low^ cells is 5.45 ± 2.9 times higher than associated total cell suspension, whereas it is only 1.43 ± 0.3 times more important for RA, demonstrating that even if both techniques allow enrichment, CSFC leads to the best one.

#### 2.2.2. KSC Sorted by Flow Cytometry (α6^high^/CD71^low^) are More Potent for Reconstructing Pluristratified Human Epidermis than RA Cells

**Figure 8:**
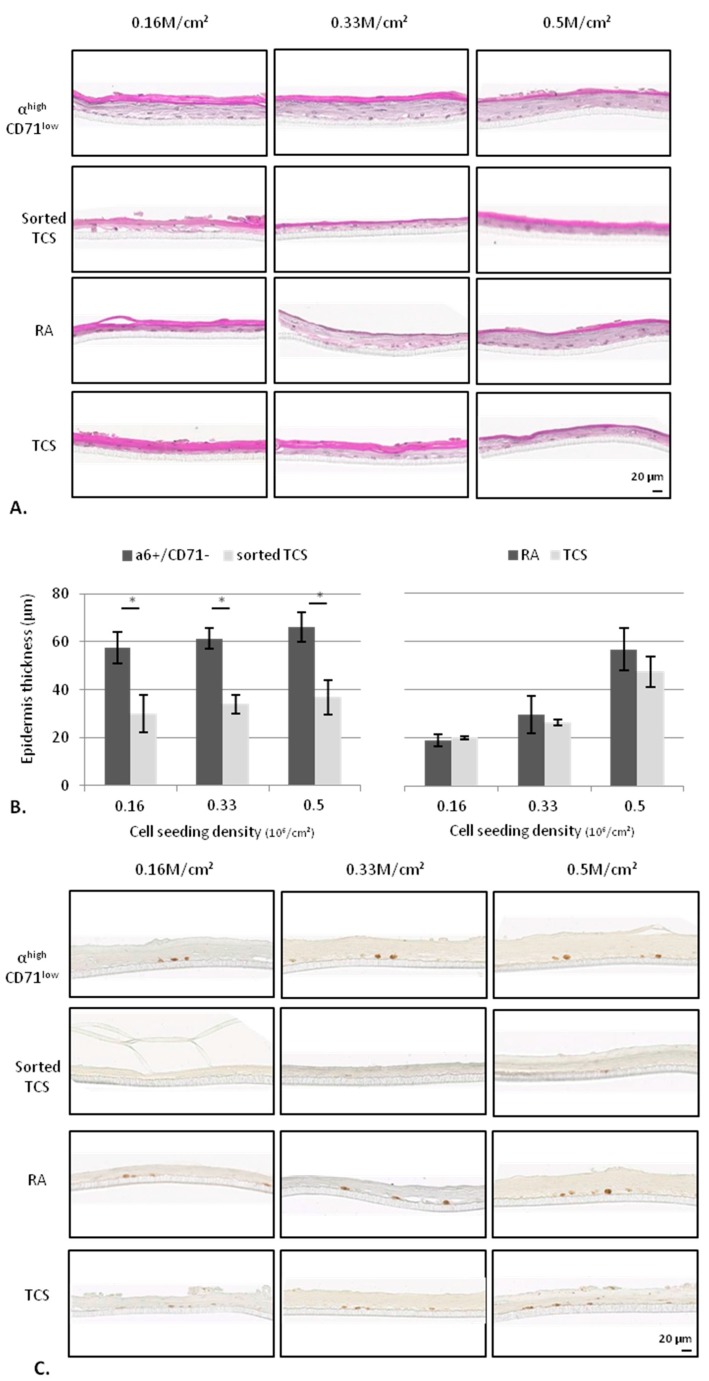

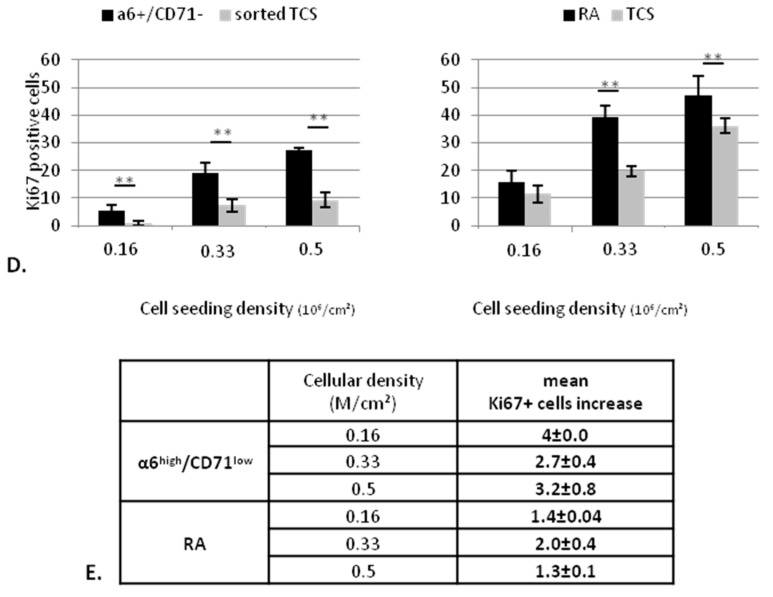
Influence of stem cell isolation method (flow cytometry compared to optimized rapid adhesion method) on capacity of cells to reconstruct human epidermis (RHE). RHE cultured with α6^high^/CD71^low^ cells and RA are compared to those cultured with corresponding sorted TCS and TCS, respectively) for morphology using hematoxylin-Phloxin-Safran staining (**A**), thickness in µM measured using ImageJ (Research Service Branch, US National Institute of Health, Bethesda, MD, USA) (**B**), and Ki67 labeling (**C**) quantified for Ki67+ cells number in whole tissue length (**D**). Representative results of two donors (**E**) Ki67+ cell number increase for both stem cell candidates (α6^high^/CD71^low^ cells and RA) compared to corresponding non-isolated cells, which are equal to 1. * data significantly different (*p* < 0.05), ** *p* < 0.005.

As Figure 8A shows, RHE exhibit between two and six living layers with *stratum granulosum* and totally differentiated *stratum corneum*. Whatever the cellular density, RHE produced with α6^high^/CD71^low^ cells appear two times thicker than those obtained with corresponding total cell suspension (TCS), whereas thickness is not statistically different for epidermis obtained with RA. Compared to RA, they are significantly thicker only for the lowest densities (0.16 and 0.33 M/cm^2^) (Figure 8B). Finally, thickness of RHE produced from α6^high^/CD71^low^ cells do not decrease with cellular density in contrast to those cultured with RA that are reduced by 49% between 0.5 and 0.33 M/cm^2^ and 35% between 0.33 and 0.16 M/cm^2^.

Concerning the proliferation state of basal cells, Figure 8C,D show that, whatever the conditions (RA, α6^high^/CD71^low^, sorted TCS and TCS), the Ki67 positive cells decrease with cellular density. The proliferative cell number is higher for RHE cultured with RA cells than those obtained from α6^high^/CD71^low^ cells for each density. However, when Ki67 positive cells are compared for each isolation technique between isolated cells (α6^high^/CD71^low^ cells or RA) and corresponding total cell suspension, it is higher for α6^high^/CD71^low^ cells than for RA, regardless of the cellular density and the donor (Figure 8E).

All together, these results show that, in accordance with previous data, α6^high^/CD71^low^ cells isolated by flow cytometry lead to a better enrichment in KSC since they result in a mature epidermis that appear thicker, with a low Ki67 expressing cell number, demonstrating an optimal balance between proliferation and differentiation.

## 3. Discussion

Our results clearly demonstrate that both rapid adhesion method and cell sorting by flow cytometry following the α6^high^/CD71^low^ phenotype (CSFC) allow enrichment in stem cells. However, despite optimization of the rapid adhesion method, CSFC leads to a better enrichment of KSC since the CFE is five times increased versus total cell suspension, whereas it is only 1.4 times for the adhesion method. These results are consistent with those observed by Kaur, who showed that cells adhered regardless of the coating (collagen I, IV and keratinocyte-secreted extra cellular matrix) and the adhesion time (from 5 to 20 min) actually corresponds to a whole α6 high population [30], as confirmed latter by Fortunel et al., who demonstrated that cells having high adhesion capacity on a type I collagen and cells with high expression of α6 integrin showed high convergence at the functional and molecular levels [21]. Our results also confirm that several markers are necessary for qualifying isolated cells as stem cells. Indeed, as no unique specific stem cell marker has been identified so far, the stem cell identity of RA cells is hardly proved and using nonspecific stem cells markers such as p63 [31,32], K15, K1/K10 [33] only shows an undifferentiated state. If Kim et al. and Gao used the α6/CD71 markers to show that RA cells are α6^high^/CD71^low^, and these results could be discussed, as all α6^high^ appeared to be CD71^low^ [11], and Gao fractioned cultured cells (2nd or 4th passage) [33]. On the other hand, we observed that these two KSC enrichment methods lead to cell populations with different responses to the isolation method. In our experience we observed that α6^high^/CD71^low^ cells require more culture days to reach the same colony size as RA cells, probably due to the stress caused by flow cytometry on sorted cells, which would need more time to enter in a mitotic program. However, this delay does not seem to have any consequences on clone-forming efficacy. Moreover, α6^high^/CD71^low^ cells lead to a thicker pluristratified epithelium with lower seeding density and display a low Ki67 positive cells number, showing that they have reached the balance between proliferation and differentiation. Consistent with our results, Schluter et al. showed that the quiescent α6^high^/CD71^low^ population was more potent for long-term epidermal renewal, from as little as 100 cells, than α6^high^/CD71^high^ population, which is very close to RA cells [34].

Concerning the optimization of the adhesion test, our data show that enrichment in KSC of a cell suspension isolated by rapid adhesion is influenced by different parameters. Indeed, by using two different models evaluating enrichment in KSC, one displaying the proportion of holoclones and meroclones directly in isolated cells (model 1) and the other defining the clonogenic potential of isolated cells (CFE obtained with the same cellular density) (model 2), this study shows that an adhesion time of 10 min on collagen I at 37 °C, and using accutase 24 h post adhesion step as a detachment method, improve KSC isolation. Indeed, model 1 demonstrates that short adhesion times allow cellular suspension richer in KSC than longer adhesion times since the percentage of holoclones decreases with the time, confirming that KSC have great adhesion capacities thanks to the surrounding niche [14,15,35], making them able to adhere quicker than TA. However, holoclones being still present in the LA fraction, removed within 10 min of adhesion, show that (i) this adhesion time could be too brief for all KSC to adhere; (ii) the particularly high cellular seeding density could lead to competition between cells; (iii) there could be an integrin gradient among KSC leading to an unequal adhesion kinetic; and (iv) the enzymatic action during the keratinocyte extraction step known to be deleterious for cell surface proteins [36,37] could have a negative influence on adhesion of some KSC.

Concerning the coating nature, no significant difference of the clonogenic and proliferative potential of RA isolated between the different coatings is observed, except the CFE obtained with fibronectin, which is significantly lower than the one observed with collagen I. Laminin, a keratinocyte adhesion molecule linking to α6β4 and α3β1 integrins and basement membrane component [38] as collagen IV [39], which is the most used and has been suggested to be involved in epidermal stem cell maintenance [40,41], appear similar to collagen I, as found in another study [30]. Moreover, no difference in cell size between colonies issued from cells isolated with fibronectin or those isolated with other coatings was observed, as it could be shown before [28]. In contrary, fibronectin leads to a significantly lower CFE than collagen I. Even if collagen I is not a part of dermo-epidermal junction, this coating globally displayed a tendency to give the best CFE for the three donors. That is why, collagen I, also being more available, was selected. This option turns out to be wise since the CFE of RA selected with collagen I was higher than with a feeder layer (2.4 ± 0.8 for collagen I and 0.8 ± 0.4 for a feeder layer), probably due to lower extra cellular matrix quantity synthesized by fibroblasts. This is confirmed by the higher holoclone number observed in RA (0.6 ± 0.3) than in LA (0.1 ± 0) with collagen, whereas they are similar after adhesion to the feeder layer. However, after collagen I adhesion, both holoclone and meroclone numbers were improved, suggesting a decrease of KSC adhesion specificity.

Concerning temperature, it is well known that reducing temperature decreases various types of cell adhesion [42,43,44,45,46,47,48], especially within the first 15 min of cell contact [42,44], which could be explained by cells viscoelastic [47,49] and deformability [50] properties, membrane anchorage formation [47] as well as membrane lipid composition and fluidity [48,51]. In consequence, our hypothesis was that low temperature adhesion could improve the specific adhesion of the most adherent cells, like KSC here. However, the best clonogenic potential is observed for RA obtained by adhesion at 37 °C.

Finally, the cell harvesting method was optimized using accutase 24 h after an adhesion step that leads to isolation of RA displaying a better clonogenic potential than immediately after adhesion or with trypsin-Ethylenediaminetetraacetic acid (EDTA). Cells may be stressed by both extraction and adhesion steps and need 24 h to recover their complete proliferative and clonogenic capacities. However, for not losing their stem cells phenotype, it is essential for RA to not enter into mitosis before their detachment. On the other hand, accutase seems to preserve “cell quality” during the detachment step since it displays a better clonogenic potential compared to those trypsinized. This is consistent with the preservation of some surface markers observed in accutase-dissociated M2 macrophage [52] and glioma stem cells [53] as well as the improvement of mouse embryonic stem cell generation [54]. This could suggest that preserving some surface molecules like integrins, strongly implied in the stem cell niche, accutase could preserve KSC phenotype during the detachment step. This hypothesis could be investigated by analyzing cell surface marker expression by flow cytometry but was not performed here since this was not the purpose of the study. Moreover, neural stem cells [55] and human embryonic stem cells’ long-term expansion are successfully performed with accutase [54]. Furthermore, we could observe that accutase does not allow cell–cell dissociation, specifying its use only for cells not developing intercellular cell junctions as in the previous examples. In our study, for cells being isolated from others and before cell division, using accutase was possible. However, performing long-term culture of accutase-dissociated keratinocyte stem cells (for several passages), as it has been shown for other types of stem cells that do not develop cell–cell junctions, was impossible.

## 4. Materials and Methods

### 4.1. Human Skin Samples

Human skin tissue explants were obtained with the informed consent of patients undergoing surgical discard, in accordance with the ethical guidelines of the Tissue Bank Institute (Edouard Herriot Hospital, Lyon, France) and the declaration to the French Research Ministry (DC No. 2008162).

### 4.2. Keratinocyte Extraction and Culture

Keratinocytes were isolated from normal human skin. Epidermis was separated from dermis using 10 mg/mL dispase (Thermo Fisher Scientific, Waltham, MA, USA). Then, keratinocytes were dissociated using trypsin EDTA 0.05% (Thermo Fisher Scientific) for 12 min at 37 °C. Cells were counted in Malassez after trypan blue 0.04% coloration. Keratinocytes were immediately seeded after extraction.

Keratinocytes were grown on feeder layers (human irradiated fibroblasts pre-seeded at 4000 cells/cm^2^) in keratinocyte medium containing Dulbecco’s Modified Eagle Medium (DMEM) and Ham’s F12 at a ratio of 3:1 (Thermo Fisher Scientific) supplemented with 10% Fetal Calf Serum (Hyclone, Logan, UT, USA), 10 ng/mL epidermal growth factor (EGF) (Sigma, St Quentin Fallavier, France), 24.3 µg/mL adenine (Sigma), 0.4 µg/mL hydrocortisone (Upjohn, Serb Laboratories, Paris, France), 0.12 IU/mL insulin (Lilly France, St Cloud, France), 2.10^−9^ M triiodo-l-thyronine (Sigma), 10^−9^ M cholera toxin (Sigma) and antibiotics. Medium was changed three times a week.

### 4.3. Colony Forming Unit and Colony Forming Efficiency

Cells were seeded at 40 cells/cm^2^ and cultured for 14 days as previously described. For clone staining, cells were fixed and colored using rhodamine (Sigma) at 0.01 g/mL in 4% paraformaldehyde for 30 min. Holoclones, meroclones and paraclones were counted. Big and homogeneous clones with regular contours are considered as holoclones. Paraclones are little and abortive colonies and meroclones are of intermediate size, heterogeneous and display irregular contours. Colony forming efficiency (CFE) was calculated as the following: CFE = CFU (meroclones + holoclones number) × 100/(cellular seeding density).

For populating doubling calculation, cells were detached using trypsin and counted. Population doubling was calculated as PD = LN (*number of harvested cells/number of cells* seeded)/LN2.

### 4.4. Experimental Protocol for Optimization of the Rapid Adhesion Method

Whatever the following steps, the experiments were performed in technical triplicates of normal keratinocytes extracted from three independent donors. The selected condition in each step was then used for the following.

Two models were used (Figure 1). Model 1 allowed an evaluation of the proportion of holoclones and meroclones directly in isolated cells (model 1). It consisted of seeding freshly extracted keratinocytes (total cell suspension TCS) at clonal density (20 cells/cm^2^). After adhesion, non-adherent or low adherent cells (LA) were removed, counted and seeded in 3 flasks containing feeder layers for CFU. Fresh medium was added to remaining adherent cells (RA). Both adherent and non-adherent cells were cultured for 14 days until the observation of colonies.

Model 2 allowed an evaluation of the clonogenic potential of isolated cells (CFE obtained with the same cellular density). It consisted of seeding freshly extracted keratinocytes (total cell suspension TCS) at high density (10^5^ cells/cm^2^). Then, after adhesion, non-adherent cells (LA) were removed and fresh medium was added to remaining adherent cells (RA). One day later, adherent cells were detached by gentle trypsinisation, counted and seeded at clonal density in 3 flasks containing feeder layers for CFU. There were cultured for 14 days until the observation of colonies.

#### Step 1: Optimization of the adhesion time using model 1

Freshly extracted keratinocytes were seeded on feeder layers. After 10 min, 30 min, 1 h or 24 h adhesion, LA were removed, counted and seeded in 3 flasks containing feeder layers for CFU. Fresh medium was added to RA. Both RA and LA were cultured for 14 days until the observation of colonies.

#### Step 2: Selection of coating using model 1 and 2

Coating selection was performed using model 2. Freshly extracted keratinocytes were seeded on several coatings known for their high cell adhesion properties: collagen I (Corning, New York, NY, USA), laminin (Corning), fibronectin (Corning), collagen IV (Corning). After 10 min (selected in previous step) of adhesion, LA were removed and fresh medium was added to RA. One day later, RA were detached with trypsin-EDTA (Sigma), counted and cultured for 14 days until the observation of colonies.

Then, fresh collagen I was compared to the feeder layer using model 1. Freshly extracted keratinocytes were seeded on collagen I. After 10 min of adhesion, LA were removed, counted and seeded in 3 flasks containing feeder layers for CFU. Fresh medium was added to RA. Both RA and LA were cultured for 14 days until the observation of colonies.

#### Step 3: Temperature selection using model 2

Freshly extracted keratinocytes were seeded on collagen I coating selected in step 2 and cultured for 10 min at 37, 22 and 4 °C. After 10 min, LA were removed and fresh medium was added to RA. One day later, RA were detached with trypsin-EDTA (Sigma), counted and cultured for 14 days until the observation of colonies.

#### Step 4: Selection of the keratinocyte detachment method using model 2

Freshly extracted keratinocytes were seeded on collagen I coating selected in step 2 and cultured for 10 min at 37 °C. After 10 min, LA were removed and fresh medium was added to RA. One day later, RA were detached with trypsin-EDTA 0.05% (Sigma) or accutase (Thermo Fisher Scientific), counted and cultured for 14 days until the observation of colonies.

### 4.5. Experimental Procedure for Comparison of Optimized Rapid Adhesion Method to FACS Sorted α6^high^/CD71^low^ Cells

From the same donor, α6^high^/CD71^low^ and RA populations were both isolated and compared for (i) CFU on three donors and (ii) their capacity to reconstruct human epidermis on two donors. For each parameter, α6^high^/CD71^low^ and RA were compared to associated total cell suspension (sorted TCS and TCS, respectively) (Figure 2).

#### 4.5.1. RA Cell Isolation by Optimized Rapid Adhesion Method (Model 2)

Briefly, freshly extracted keratinocytes were seeded on collagen I for 10 min (RA) at 37 °C and LA were removed. Fresh medium was added to RA. One day later, RA were detached using accutase, counted and cultured for CFU and RHE.

#### 4.5.2. KSC Isolation by Flow Cytometry

Freshly extracted keratinocytes were incubated in saturation solution composed of bovine serum 4% in PBS for 15 min at 4 °C. Cells were incubated with antibodies anti CD71/transferrin receptor (BD biosciences, Franklin Lakes, NJ, USA) for 30 min at 4 °C. After washing, cells were stained with R-Phycoerythrin (R-PE)-conjugated rat anti-human CD49f antibody (mAb) and streptavidin allophycocyanin (SAV-APC) for 30 min at 4 °C. For each sort, an appropriate isotype-control was used to remove the level of background signal. Both α6^high^/CD71^low^ (KSC) and α6^high^/CD71^high^ (TA) populations were isolated using a MoFlo cell sorter (DakoCytomation, Glostrup, Denmark). After sorting, cells were counted and viability was controlled using trypan blue before seeding for CFU and RHE.

### 4.6. Epidermis Reconstruction

#### 4.6.1. Preparation

Cells were seeded at different densities (0.5, 0.33, 0.16 M/cm^2^) in culture inserts 0.4 µm PCF (Millicell, Millipore, Darmstadt, Allemagne). Cells were cultured in keratinocyte medium for 7 days in immerged conditions. Then, cultures were lifted at air/liquid interface for 7 additional days in differentiation medium (DMEM supplemented with 8 mg/mL bovine serum albumin (Sigma), 0.12 IU/mL insulin, 0.4 µg/mL hydrocortisone, and antibiotics).

#### 4.6.2. Analysis by Histology and Immunohistological Analysis

Reconstructed epidermis were fixed in neutral buffered formalin 4% (Diapath, Martinengo BG, Italia) for 24 h, embedded in paraffin and cut into 5 μm sections. They were dewaxed and rehydrated, and then stained with haematoxylin-phloxin-saffron (HPS staining).

For immunohistological analysis, endogenous peroxydases were inactivated by incubating sections in 5% H_2_O_2_/3% normal goat serum (NGS; Jackson Immunoresearch, West Grove, PA, USA). Incubation in PBS containing 5% of Normal Goat Serum (NGS) for 45 min was performed for blocking non-specific binding. Then, primary antibodies diluted in PBS/NGS 5% were added overnight at room temperature: Ki67 (Dako, LES ULIS, France). Secondary HRP-anti-mouse (Dako) was incubated for 1 h at room temperature. Labelling was revealed using DAB (Dako) and slides were then stained with hematoxilin.

The software MBF_ImageJ for microscopy (Research Service Branch, US National Institute of Health, Bethesda, MD, USA) was used for image processing and analysis. Epidermal thickness was measured as the distance between the basal layer and the last living layer of the epidermis in 40 different fields per sample, and the number of Ki67 positive-cells was counted in whole length samples for the proliferative state (*n* = 3).

### 4.7. Statistical Analysis

For all data, the statistical significance was assessed on three donors running Mann–Whitney tests by using the software GraphPad Prism 4 (GraphPad Software Inc., La Jolla, CA, USA), and differences are considered statistically significant for *p* ≤ 0.05.

## 5. Conclusions

To conclude, even with the best conditions for isolating epidermal stem cells by the rapid adhesion method, RA are not the same population as KSC isolated by flow cytometry following α6^high^/CD71^low^ phenotype. Unfortunately, several studies have attempted to analyze the KSC response to several genotoxic agents by using the rapid adhesion technique [56,57]. However, our study shows clearly that the RA cells are much closer to the progenitor cells than the stem cells. Finally, the sorted α6^high^/CD71^low^ population will be very a valuable model to study the response of KSC to several chemical or physical stresses or to screen molecules capable of protecting KSC and keeping their regenerative capacities.

## Abbreviations

KSC	keratinocyte stem cells
RA	rapid adherent cells
LA	low adherent cells
TCS	total cell suspension
RHE	reconstructed human epidermis
CFU	colony forming unit
CFE	colony forming efficiency
